# Antibodies Against Unusual Forms of Sialylated Glycans

**DOI:** 10.32607/actanaturae.11631

**Published:** 2022

**Authors:** P. S. Obukhova, M. M. Ziganshina, N. V. Shilova, A. A. Chinarev, G. V. Pazynina, A. Y. Nokel, A. V. Terenteva, N. R. Khasbiullina, G. T. Sukhikh, A. A. Ragimov, E. L. Salimov, V. I. Butvilovskaya, S. M. Polyakova, J. Saha, N. V. Bovin

**Affiliations:** Shemyakin-Ovchinnikov Institute of Bioorganic Chemistry of the Russian Academy of Sciences, Moscow, 117997 Russia; National Medical Research Center for Obstetrics, Gynecology and Perinatology named after V.I. Kulakov of the Ministry of Health care of Russian Federation, Moscow, 117997 Russia; I.M. Sechenov First Moscow State Medical University of the Ministry of Health care of the Russian Federation (Sechenov University), Moscow, 119991 Russia; Engelhardt Institute of Molecular Biology of the Russian Academy of Sciences, Moscow, 119991 Russia; Synthaur LLC, Moscow, 117997 Russia; Centre of Biomedical Research, Sanjay Gandhi PostGraduate Institute of Medical Science, Lucknow, 226014 India; Centre for Kode Technology Innovation, Auckland University of Technology, Auckland, 1010 New Zealand

**Keywords:** alylated glycans, Kdn, human natural antibodies, pregnancy, glycoarray

## Abstract

Previous studies have shown that in the blood of healthy donors (1) there are no natural antibodies against sialylated glycoproteins, which contain Neu5Acα (N-acetylneuraminic acid) as the most widespread form of human sialic acid, and (2) there is a moderate level of antibodies capable of binding unnatural oligosaccharides, where Neu5Ac is beta-linked to a typical mammalian glycan core. In the present study, we investigated antibodies against βNeu5Ac in more detail and verified the presence of Kdn (2-keto-3-deoxy- D-glycero-D-galacto-nonulosonic acid) as a possible cause behind their appearance in humans, taking into account the expected cross-reactivity to Kdn glycans, which are found in bacterial glycoconjugates in both the α- and β-forms. We observed the binding of peripheral blood immunoglobulins to sialyllactosamines (where “sialyl” is Kdn or neuraminic acid) in only a very limited number of donors, while the binding to monosaccharide Kdn occurred in all samples, regardless of the configuration of the glycosidic bond of the Kdn moiety. In some individuals, the binding level of some of the immunoglobulins was high. This means that bacterial Kdn glycoconjugates are very unlikely to induce antibodies to βNeu5Ac glycans in humans. To determine the reason for the presence of these antibodies, we focused on noninfectious pathologies, as well as on a normal state in which a significant change in the immune system occurs: namely, pregnancy. As a result, we found that 2/3 of pregnant women have IgM in the blood against Neu5Acβ2-3Galβ1-4GlcNAcβ. Moreover, IgG class antibodies against Neu5Acβ2-3Galβ1-4GlcNAcβ and Neu5Acβ2-6Galβ1-4GlcNAcβ were also detected in eluates from the placenta. Presumably, these antibodies block fetal antigens.

## INTRODUCTION

Within animal cells, sialic acids (N-acetylneuraminic acid, Neu5Ac, and N-glycolylneuraminic acid, Neu5Gc, the two most abundant forms) in glycoproteins are normally found at the terminal positions of complex glycans linked by α2,3 or α2,6 to Gal or α2,6 to GalNAc penultimate residues. Sialylated glycans are involved in numerous biological recognition processes [1]; so, it is not surprising that antibodies (i.e., autoantibodies) against Neu5Acα-glycans are not found in healthy individuals [2]. However, antibodies against some oligosaccharides terminated by β-form Neu5Ac, not found in nature, have been documented [2]. To explain their origin and predetermination, it has been hypothesized that formal antibodies against βNeu5Ac actually target fragments of bacterial polysaccharides/lipopolysaccharides of *Streptomyces*, *Klebsiella*, etc. (according to http://csdb.glycoscience.ru/database/), which often contain structurally similar sialic acid; namely, the β-anomer of 2-keto-3-deoxy-D-glycero-D-galacto- nonulosonic acid (Kdn) [2, 3]. In support of this idea there was the presence of antibodies in healthy donor blood against Kdn-glycans typical of the lipopolysaccharide core motif [4]. Therefore, we investigated human antibodies against Kdn glycans using synthetic spacer-armed α- and β-Kdn monosaccharides [5], Kdn-form of 6’- and 3’-sialyllactosamines, as well as the corresponding Neu5Acα- and β-derivatives in parallel (*Scheme*), by immobilizing them together with other glycans on a microchip. Blood sera from healthy donors, healthy pregnant women, and women with complicated pregnancies were studied using this “sialic” printed glycan array (sialic PGA). In this article, we discuss the possible causes of the emergence of antibodies directed to the β-form of sialic acids.

**Scheme:**
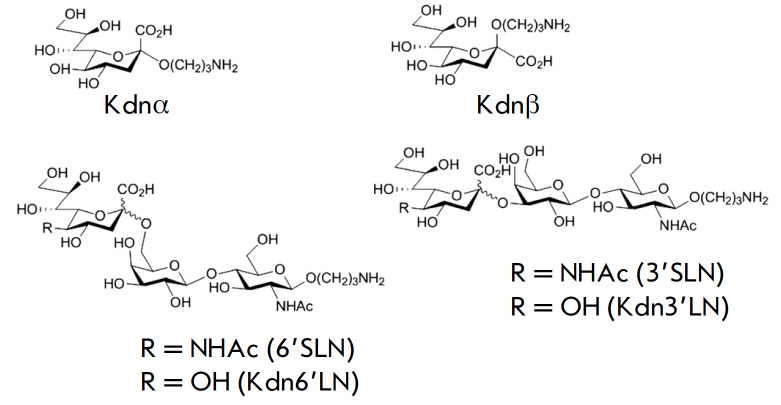
The structure of the synthetic Kdn-glycans used in this work (in the composition of sialic PGA) and parent Neu5Ac-trisaccharides

## EXPERIMENTAL

**Kdn-glycans**

Kdn-glycans were obtained [5] as individual anomers (of 95% purity according to HPLC and NMR), and their anomeric stereochemistry was determined based on chemical shifts and coupling constants for Kdn H-3 protons, as previously described [5].

**Donors and patients**

**Table 1:** Samples and corresponding versions of glycochips

Cohorts		Number of individuals	Biomaterials	Number of samples	PGA version	Sources of biomaterials
1	Healthy donors	16	Blood sera	16	#1 sialic PGA (17 oligosaccharides)	I.M. Sechenov First Moscow State Medical University of the Ministry of Health care of the Russian Federation (Sechenov University), Moscow, 119991 Russia
2	Healthy fertile women (donors)	26	Blood sera	26	#3 (441 oligosaccharides and 219 bacterial polysaccharides)	National Medical Research Center for Obstetrics, Gynecology and Perinatology named after V.I. Kulakov of the Ministry of Health care of Russian Federation, Moscow, 117997 Russia
3	Healthy pregnant women	30	Blood sera	26	#2 (381 oligosaccharides)	
			Eluates from placentas	30		
4	Patients with pregnancy complications	32	Blood sera	29		
			Eluates from placentas	32		

We examined biological samples from 104 individuals. The original cohort (Group 1), which consisted of 16 donors (8 women and 8 men) from I.M. Sechenov First Moscow State Medical University (Moscow, Russia), and a retrospective cohort consisting of 88 patients from the National Medical Research Center for Obstetrics, Gynecology and Perinatology (Moscow, Russia) met the inclusion criteria and were selected for participation in the study (*Table 1*). In the retrospective cohort, 26 healthy nonpregnant women applied to a pregnancy planning center (Group 2); 30 patients with normal pregnancies (Group 3); and 32 patients with complicated pregnancies (Group 4), including 41% with preeclampsia (PE), 25% with fetal growth restriction (FGR), and 34% with PE accompanied by FGR. The inclusion criteria for Group 1 were as follows: age greater than or equal to 18, the absence of absolute contraindications for donation, normal blood tests, biochemical blood analysis, coagulogram and blood pressure. The inclusion criteria for Group 2 were as follows: more than one pregnancy, occurring in the natural cycle without assisted reproduction technology, normal menstrual cycle and absence of hormonal dysregulation. Inclusion criteria for Group 3 were the absence of any chronic gynecological or somatic disease, no threat of abortion, early toxicosis, inflammatory disease, PE or FGR, no medical therapy (except for vitamins or mineral supplements), normal vaginal flora, and normal ultrasonography and Doppler ultrasonography during current pregnancy. The inclusion criteria for Group 4 were pregnancy complicated by PE, and/or FGR. All pregnant women had spontaneous singleton pregnancies and gave birth by cesarean section. Pregnant women with the HELLP syndrome (an atypical form of severe preeclampsia, which is characterized by symptoms: H – hemolysis, EL – elevated liver enzymes, LP – low platelet count in the blood) were excluded from the study. The exclusion criteria for all groups were severe somatic diseases, including autoimmune diseases, acute and chronic inflammatory diseases, acute and chronic inflammatory diseases in the acute stage, a history of blood transfusion or organ transplantation, immunotherapy, hormone therapy, and the use of drugs that affect antibody production and bioavailability, including low-molecular-weight heparins. All subjects provided written informed consent before participation. The study protocol was approved by the local ethical committee of the related medical organizations.

**Diagnostic evaluation of pregnancy disorders**

**Table 2:** Clinical characteristics of the study groups

Features	Group 1 healthy donors	Group 2 nonpregnant healthy women	Group 3 women with normal pregnancy	Group 4 women with complicated pregnancy	p value*
Age (years)**	33.0 (18–62)	30.0 (24–44)	32.5 (23–40)	34.5 (24–45)	0.2253
Systolic arterial blood pressure (mm Hg)**	–	118.0 (110–120)	110 (103–130)	150.0 (110–210)	< 0.0001
Diastolic arterial blood pressure (mm Hg)**	–	75.0 (70–82)	70.0 (60–80)	95.0 (70–115)	< 0.0001
Newborns’ gestational age at delivery (weeks)***	–	–	39.2 (39.0–40.0)	34.8 (30.40–37.20)	< 0.0001
Newborns’ weight, g**	–	–	3462.0 (2800–4180)	1997.0 (440–3300)	< 0.0001
Newborns’ Apgar Scores**	–	–	8.0 (8)	7.0 (2–8)	< 0.0001

^*^Groups 3 and 4 comparison.

^**^data are presented as a median with min. and max. values, Mann–Whitney test.

^***^data are presented as a median with interquartile range, Mann–Whitney test.

Patients were included in the groups according to the criteria of the International Society for the Study of Hypertension in Pregnancy (ISSHP) [6]. The prenatal diagnosis of FGR was based on the criteria described in [7]. The clinical characteristics of the study groups are shown in *Table 2*.

**Blood serum sample collection**

In the original cohort (Group 1), serum samples were collected in vacuum blood collection tubes VACUETTE® Serum, cap red, with clotting activator and gel for separation (4 ml, L×Ø =75 × 13 mm). In the retrospective cohort, serum samples were collected in vacuum blood collection tubes S-Monovette® Serum, cap white, with clotting activator (4.9 ml, L×Ø = 90 × 13 mm). Within 1 h of blood collection, the samples were centrifuged for 10 min at 2,000 *g* and stored at −80°C until antibody analysis.

**Elution of antibodies from the placenta**

The placenta was obtained from patients in Groups 3 and 4 during cesarean section, and placenta-associated antibodies were eluted, as previously described [8], using 10.0 g of placental tissue (mainly the villous chorion, basal, and chorial lamina were taken). An equal amount of placental tissue was collected from each patient. Samples of eluates with the SIGMAFAST protease inhibitor (S8820, Sigma– Aldrich, MO, USA) at the concentration recommended by the manufacturer were stored for a maximum of 7 days at 4°C before the analysis. Analysis of the eluted antibodies was performed using PGA as described below, with only IgG detection. Placental eluates were applied to the glycochip without dilution, and the concentration of the eluted antibodies was standardized by means of the same amount of placental tissue and identical elution procedures.

**PGA assay**

Glycochips of three formats were used (Semiotik LLC, Russia): #1 – containing only sialylated glycans (approximately 20 glycans, this version of the glycochip was called “sialic PGA”), #2 – containing 381 oligosaccharides, #3 – containing 441 oligosaccharides and 219 bacterial polysaccharides; the second and third versions included all the sialylated glycans of the first. The purity of the glycans was 95-98%, according to HPLC and NMR data. Glycan printing was carried out in accordance with international rules, as previously described [4]. Each ligand on the array was applied in 6-12 repeats; ligand immobilization on the array was monitored using human serum, monoclonal and affine-purified polyclonal antibodies and plant lectins according to the manufacturer’s quality control protocol.

The analysis of blood sera and the eluates from the placenta using PGA was performed as previously described [4, 8]. The correspondence of the samples and formats of the glycochips is presented in *Table 1*. Signals were measured as the medians of relative fluorescence units (RFU) for replicates with median absolute deviations. The background value was determined as the signal from the ligand-free spot. The background value multiplied by a factor of 10 was taken as the cutoff. Signals above the cutoff were considered significant. The frequency of the specific anti-glycan antibody occurrence was calculated as a percentage (%) of the number of individuals in whom the median RFU for the corresponding glycan as a result of blood serum analysis in PGA was higher than the cutoff.

**Statistical analysis**

The Mann–Whitney U test was performed for intergroup comparison using the MedCalc software version 16.4 (MedCalc, Belgium). Differences were considered significant if the *p *value was below 0.05.

## RESULTS AND DISCUSSION

2-Keto-3-deoxy-D-glycero-D-galacto-nonulosonic acid (Kdn) is found in noticeable amounts in bacteria and ectothermic vertebrates. From a molecular perspective, these are glycolipids, glycoproteins, bacterial capsular polysaccharides, and bacterial lipopolysaccharides, where Kdn is linked as a 2,3-, 2,4-, 2,6- or 2,8-substituent. In humans, Kdn has been found in very small amounts; 0.1–1% of total sialic acid, in all types of glycoconjugates in various organs [9]. It is believed that Kdn enters the human metabolic system from the outside through food, similar to Neu5Gc [10]; slightly increased Kdn expression was found in human fetal red blood cells compared to adult cells and in ovarian tumor tissues [9]. Kdn is widespread in organisms with which humans come into contact and is definitely an alien monosaccharide to humans, even in its α-linked form.

**Fig. 1:**
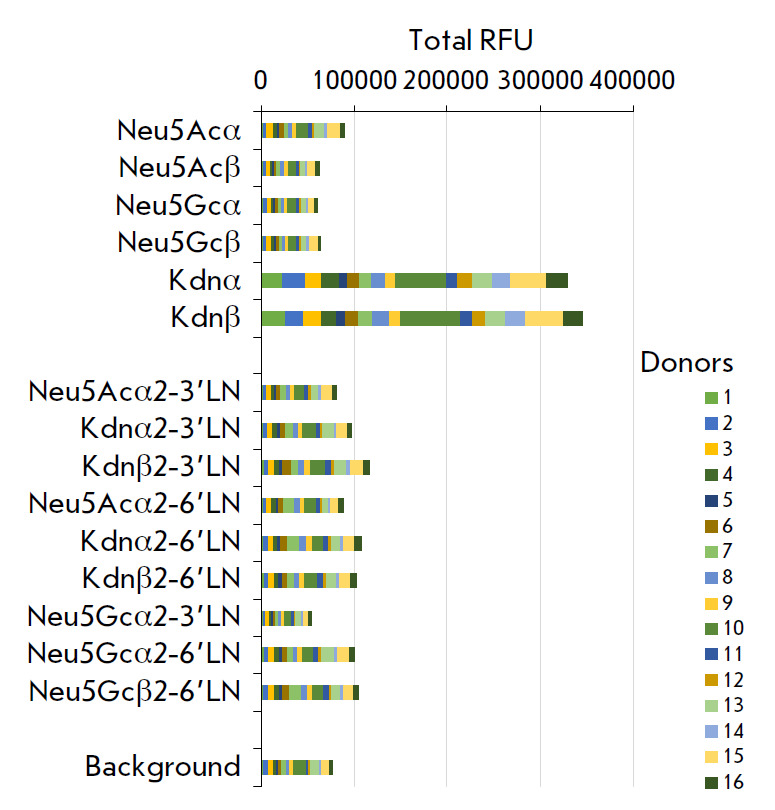
Binding of human IgG antibodies (sera from 16 donors) to sialylated glycans. The sialic PGA (format #1) data are presented as a stacked chart. The RFU values for all donors are summarized

When testing 16 blood serum samples from healthy donors (Group 1) using sialic PGA, we did not detect IgG antibodies against either Kdnα2- 3Galβ1-4GlcNAcβ (Kdnα2-3’LN) or Kdnα2-6Galβ1- 4GlcNAcβ(Kdnα2-6’LN), or against their corresponding Kdnβ-versions (*Fig. 1*).

**Fig. 2:**
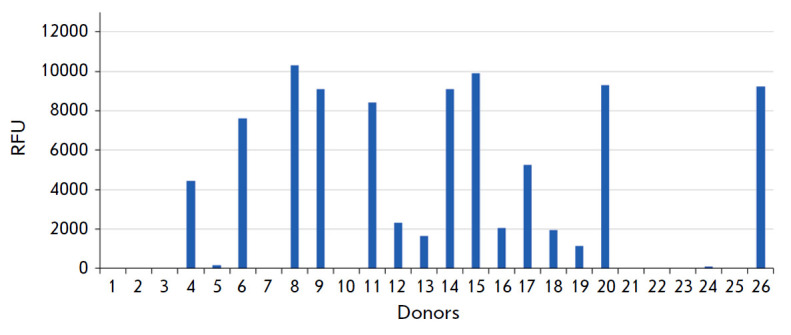
Fifteen out of twenty-six healthy donors had antibodies (IgM) against the trisaccharide Kdnα2-3Galβ1-4GlcNAcβ. The data of the PGA of format #3 are presented. The cutoff value was subtracted

However, in this small cohort, we did not observe antibodies against the α-linked form of Kdn, Kdnα2- 3Galβ1-4GlcNAc, although A. Varki’s group detected IgG antibodies against it in a limited number of donors using a similar PGA [10, 11]. To address this inconsistency, we extracted data from our archives corresponding to the data for contingently healthy women, where the full version of PGAs was used, which included the trisaccharide Kdnα2-3Galβ1- 4GlcNAc. According to these data, compiled in *Fig. 2*, 15 out of 26 donors did have antibodies against the trisaccharide, but this only applies to IgM, as IgG antibodies were not detected. Information about the samples studied and the corresponding versions of the PGA is given in *Table 1*.

We believe the discrepancy above between the results is due to the fact that antibodies capable of binding to the Kdn-form of sialyllactosamine are formed in response to bacterial infections (i.e., we deal with adaptive immunoglobulins), which emerge with different frequencies in different small-sized cohorts, depending on the region, season, etc.

**Fig. 3:**
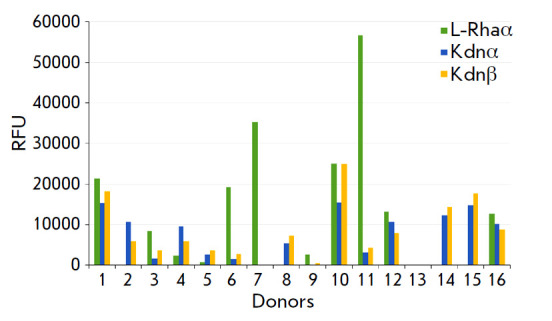
Binding of human IgG-class serum antibodies (from 16 donors) to Kdn monosaccharide in its α- and β-spacer form compared to αL-rhamnose (L-Rhaα). The sialic PGA (format #1) data are presented. The cutoff value was subtracted

In contrast to antibodies directed to Kdntrisaccharides, a moderate (or sporadically high) level of IgG antibodies against the monosaccharide Kdn was observed in most donors (*Fig. 3*), interestingly, almost indentical for both the α- and β-form.

The observed RFU values for the Kdn monosaccharide in highly responsive donors were close to the values for L-rhamnose, which is used here as a high binding reference, while no antibodies were found against the Neu5Ac monosaccharide (both α- and β-) (*Fig. 1*), which is confirmed by previously published data [4]. Apparently, the immunoglobulins that bind to the monosaccharide Kdn broadly recognize antibodies against bacterial polysaccharides, to the main chain of which Kdn is attached as a pendant residue.

The absence of antibodies to trisaccharides, in which the Kdn residue is linked by a β-glycosidic bond, indicates that Kdn-containing lipopolysaccharides are unlikely to trigger the appearance in humans of the previously observed (see above) antibodies against glycans containing β-linked N-acetylneuraminic acid.

However, anti-Kdn antibodies were not the subject of this study. In this study, a critical issue for us was to explain the origin and biological significance of previously identified antibodies against Neu5Acβ- glycans [2]. As noted above, the assumption that lipopolysaccharides are a trigger of and target for them is inconsistent with new experimental data; namely, the absence of any evidence of their binding to Kdnlactosamines in the overwhelming number of donors and the inability to distinguish between the α- and β-forms of the monosaccharide Kdn. Therefore, an alternate explanation for the origin and function of antibodies to Neu5Acβ-glycans is warranted. Our first attempt at this is outlined below.

**Table 3:** The occurrence frequency of the corresponding antibodies recognizing sialylated glycans (this parameter is a % of individuals whose RFU was higher than cutoff)

Sialylated glycans	% in women with normal pregnancy (blood)		% in women with complicated pregnancy (blood)		% in women with normal pregnancy (placenta)	% in women with complicated pregnancy (placenta)
Structure	IgG	IgM	IgG	IgM	IgG	IgG
Neu5Acβ2-3Galβ1-4GlcNAcβ (β3’SLN)	4	65	0	69	30	19
Neu5Acα2-3Galβ1-4GlcNAcβ (3’SLN)	0	15	0	35	0	0
Neu5Acβ2-6Galβ1-4GlcNAcβ (β6’SLN)	19	31	7	45	20	3
Neu5Acα2-6Galβ1-4GlcNAcβ (6’SLN)	0	0	0	17	27	6

**Fig. 4:**
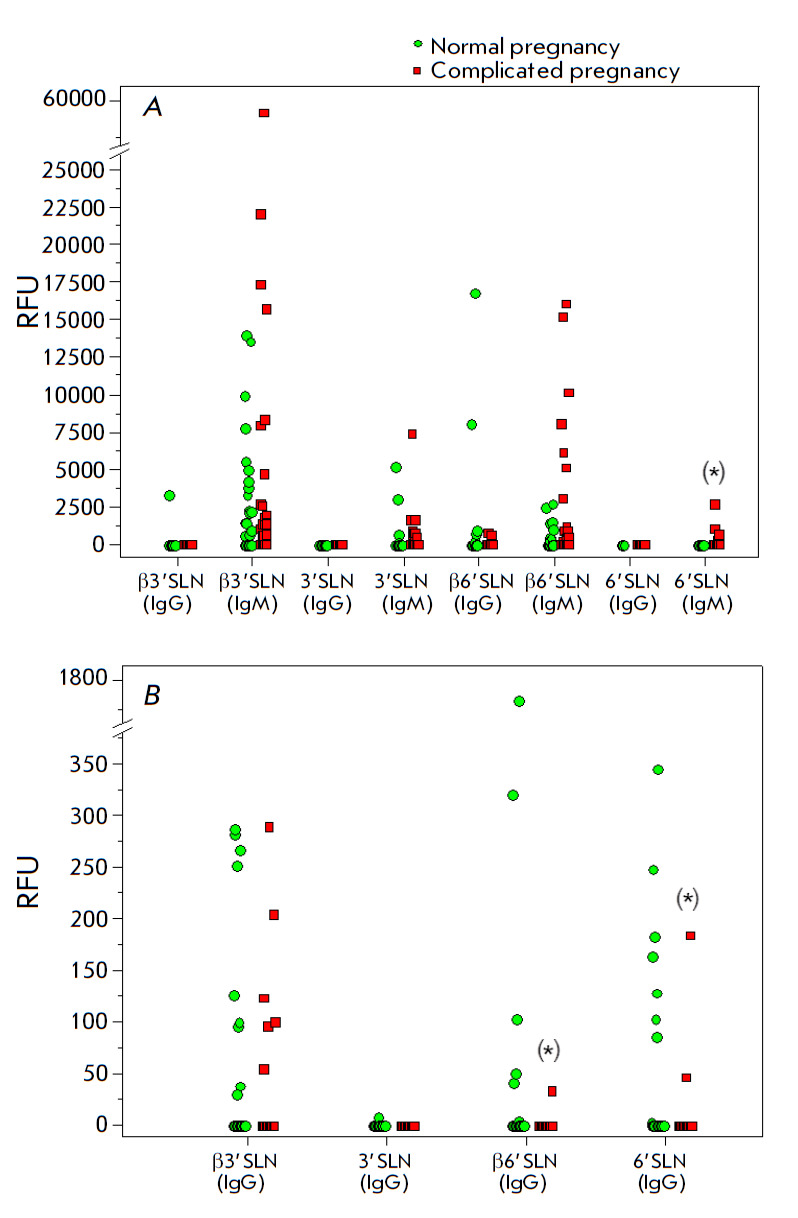
Binding of serum IgG and IgM antibodies (A) and eluated placenta-associated IgG antibodies (B) to 3’SLN and 6’SLN trisaccharides (comparison of their Neu5Acβ- vs. Neu5Acα-forms). The data of the PGA of format #2 for 30 healthy pregnant women and 32 women with pregnancy complications (preeclampsia and fetal growth restriction). The cutoff value was calculated for each group separately (for sera and eluates, for IgG and IgM) as described in the Materials and Methods section. (*) - the intergroup difference was significant (U test, p < 0.05)

We investigated how often antibodies against Neu5Acβ2-3Galβ1-4GlcNAcβ and Neu5Acβ2-6Galβ1- 4GlcNAcβ (β-forms of 3′SLN and 6′SLN) in the blood of healthy pregnant women are encountered, as well as how often they are encountered in the blood of patients with pregnancy complications caused by PE and FGR. These complications are the great obstetrical syndromes associated with the disorders of deep placentation and impaired immune response to alloantigens [12, 13]. In addition to blood antibodies, eluates from the placentas of Groups 3 and 4 were also examined (*Table 3, Fig. 4*).

The observed frequency was surprisingly high (*Table 3, Fig. 4*), especially for the occurrence of antibodies against the β2-3 isomer of SLN, Neu5Acβ2- 3Galβ1-4GlcNAcβ. As mentioned above [2, 14], antibodies against the corresponding α-sialylated glycans were practically absent in healthy subjects. They were rarely found in women with normal pregnancies and were found more frequently in individuals with pregnancy complications, which are apparently associated with a general impaired immune response [15] and impaired tolerance of the fetus. New data have confirmed this observation. Notably, antibodies against Neu5Acβ-glycans were also detected quite often (20-30% of cases) in eluates from the placenta (*Table 3*). Indeed, this is only IgG, since IgM is absent in the placenta. At the same time, anti-Neu5Acβ2- 3Galβ1-4GlcNAcβ IgM antibodies were found with a high frequency in the blood of these patients whereas IgG antibodies with this specificity were absent in their blood. Since the antigens of both parents may be present in the placenta [16], we assume that these placental immunoglobulins G located in resident in the placenta and found in eluates play the role of protectors against the maternal immune system by binding to alloantigens in the placenta. This is supported by their lower incidence in patients with complicated pregnancies. Apparently, in PE and FGR, the mechanism of masking alloantigens by placental antibodies is impaired. This assumption is consistent with the concept that the production of other protective antibodies during pregnancy masks fetal alloantigens in the placenta from an attack by the mother’s immune system [17, 18]. J. Gu et al. [19] demonstrated that protective IgG antibodies are generated by placental cells and regulate local immune reactions. The second (and more plausible in our opinion) explanation for the fact that antibodies are found in the placental tissue but are absent in the peripheral blood is their complete - or almost complete - harboring on the placental antigens, as a result of which their content in the blood drops below the sensitivity threshold of the detection method. While there are no direct data available, we believe that for the observed antibodies, β-sialosides are mimotopes of protein antigens. Identifying the true epitopes is the next challenge.

## CONCLUSIONS

The profiling of human antibodies using a comprehensive glycan array reveals a number of immunoglobulins with unexpected specificities, which include antibodies to β-linked sialic acid. The search for what is behind the presence and function of these antibodies was the aim of this study. We assumed that the identified antibodies are directed to Kdn-containing glycoconjugates of bacterial origin, which occur in both α- and β-linked forms. However, this hypothesis is not supported by the new data presented here; that is, the true target antigens and the physiological role of these antibodies have yet to be determined. At the same time, we found antibodies in the blood (IgM) and placental tissue (IgG) of pregnant women, which provides grounds for searching for a physiological role for antibodies to the β-form of sialic acid (or its antigen-mimetic) in reproductive immunology

## ACKNOWLEDGMENTS

The experiments were partially carried out using equipment provided by the Shemyakin-Ovchinnikov Institute of Bioorganic Chemistry (IBCh) core facility (CKP IBCh, supported by the Russian Ministry of Education and Science [agreement No. RFMEFI 621117X0018]). The authors wish to thank the Laboratory for Collection and Storage of Biological Material (Biobank) of the National Medical Research Center for Obstetrics, Gynecology and Perinatology named after V.I. Kulakov for providing the blood samples.

## Abbreviations

FGR: fetal growth restriction
Gal: galactose
GalNAc: N-acetylgalactosamine
GlcNAc: N-acetylglucosamine
Kdn: 2-keto-3-deoxy-D-glycero-D-galacto-nonulosonic acid
LN: N-acetyllactosamine Galβ1-4GlcNAcβ
Neu5Ac: N-acetylneuraminic acid
Neu5Gc: N-glycolylneuraminic acid
PE: preeclampsia
PGA: printed glycan array
RFU: relative fluorescent units
SLN: sialyllactosamine

## References

[1] Varki A. (2007). Nature.

[2] Shilova N., Huflejt M.E., Vuskovic M., Obukhova P., Navakouski M., Khasbiullina N., Pazynina G., Galanina O., Bazhenov A., Bovin N. (2015). Top. Curr. Chem..

[3] Deng L., Chen X., Varki A. (2013). Biopolymers..

[4] Obukhova P., Tsygankova S., Chinarev A., Shilova N., Nokel A., Kosma P., Bovin N. (2020). Glycobiology..

[5] Chinarev A.A., Sablina M.A., Kunetskiy R.A., Shilova N.V., Polyakova S.V., Paramonov A.S., Saha J., Bovin N.V. (2021). Mendeleev Commun..

[6] Brown M.A., Magee L.A., Kenny L.C., Karumanchi S.A., McCarthy F.P., Saito S., Hall D.R., Warren C.E., Adoyi G., Ishaku S. (2018). Pregn. Hypert..

[7] Ziganshina M.M., Kulikova G.V., Fayzullina N.M., Yarotskaya E.L., Shchegolev A.I., Le Pendu J., Breiman A., Shilova N.V., Khasbiullina N.R., Bovin N.V. (2020). Placenta..

[8] Ignat’eva N.V., Ziganshina M.M., Shilova N.V., Khasbiullina N.R., Bovin N.V., Tyutyunnik V.L., Sukhikh G.T. (2019). Bull. Exp. Biol. Med..

[9] Inoue S., Kitajima K. (2006). Glycoconj. J..

[10] Kawanishi K., Saha S., Diaz S., Vaill M., Sasmal A., Siddiqui S.S., Choudhury B., Sharma K., Chen X., Schoenhofen I.C. (2021). J. Clin. Invest..

[11] Saha S., Coady A., Sasmal A., Kawanishi K., Choudhury B., Yu H., Sorensen R.U., Inostroza J., Schoenhofen I.C., Chen X. (2021). mBio..

[12] Brosens I., Pijnenborg R., Vercruysse L., Romero R. (2011). Am. J. Obstet. Gynecol..

[13] Wilczynski J.R. (2006). Hum. Immunol..

[14] Huflejt M.E., Vuskovic M., Vasiliu D., Xu H., Obukhova P., Shilova N., Tuzikov A., Galanina O., Arun B., Lu K. (2009). Mol. Immunol..

[15] Yang X., Zhang C., Chen G., Sun C., Li J. (2019). J. Obstet. Gynaecol. Res..

[16] Deshmukh H., Way S.S. (2019). Annu. Rev. Pathol..

[17] Barrientos G., Fuchs D., Schrocksnadel K., Ruecke M., Garcia M.G., Klapp B.F., Raghupathy R., Miranda S., Arck P.C., Blois S.M. (2009). J. Reprod. Immunol..

[18] Malan Borel I., Gentile T., Angelucci J., Pividori J., Guala M.C., Binaghi R.A., Margni R.A. (1991). J. Reprod. Immunol..

[19] Gu J., Lei Y., Huang Y., Zhao Y., Li J., Huang T., Zhang J., Wang J., Deng X., Chen Z. (2015). Hum. Reprod..

